# Investigation of the Relationship between Work Ability and Work-related Quality of Life in Nurses

**Published:** 2017-10

**Authors:** Milad ABBASI, Abolfazl ZAKERIAN, Arash AKBARZADE, Nader DINARVAND, Maryam GHALJAHI, Mohsen POURSADEGHIYAN, Mohammad Hossein EBRAHIMI

**Affiliations:** 1. Research Center for Environmental Determinants of Health (RCEDH), Kermanshah University of Medical Sciences, Kermanshah, Iran; 2. Student’s Scientific Research Center, Tehran University of Medical Sciences, Tehran, Iran; 3. Dept. of Occupational Health, School of Public Health, Tehran University of Medical Sciences, Tehran, Iran; 4. Dept. of Epidemiology & Biostatistics, School of Public Health, Tehran University of Medical Sciences, Tehran, Iran; 5. Dept. of Nursing, School of Nursing and Midwifery, Tehran University of Medical Sciences, Tehran, Iran; 6. Dept. of Occupational Health, School of Public Health, Zabol University of Medical Sciences, Zabol, Iran; 7. Dept. of Ergonomics, School of Rehabilitation, University of Social Welfare and Rehabilitation Sciences, Tehran, Iran; 8. Occupational and Environmental Health Research Center, Shahroud University of Medical Sciences, Shahroud, Iran

**Keywords:** Nurses, Quality of working life, WAI questionnaire, Work ability, WRQoL scale

## Abstract

**Background:**

Work ability of nurses is an index of their job satisfaction and is a crucial factor in job quality and security. This study aimed to investigate the association between work ability and quality of working life and to determine the effective demographic and background variables, among nurses.

**Methods:**

The present study was conducted among nurses, working in educational hospitals under the supervision of Tehran University of Medical Sciences in 2014. Work Ability Index (WAI) and Work-related Quality of Life (WRQoL) were used.

**Results:**

The mean WAI was significantly associated with total WRQoL score and the two of its sub-items including Stress at Work, and General Well-Being (*P*-value=0.001). Moreover, the results showed a significant correlation between total WRQoL and WAI Subscales including mental resources (*P*-value=0.001), number of current diseases (*P-*value=0.02), and work ability in relation to the job demands (*P*-value=0.04). The WRQoL and WAI showed significant associations with age and job experience (*P*-value=0.001). The average score of WAI and WRQoL was statistically different among various working units (*P*-value=0.001).

**Conclusion:**

Overall, results support the association between nurses work ability and WRQoL. Monitoring the WRQoL and work ability of employees would help organizations to know their status and take measures to ameliorate the working conditions.

## Introduction

Nurses are considered as the integral part of health care system (1). However, nursing work-force is exposed to high level of occupational stress and various psychological problems (1, 2). Nurses’ poor mental health has detrimental effects not only on themselves but also on the patients under their supervision (3). What is more, a global pressure is on the healthcare systems due to a mismatch in demand and supply. The shortage of qualified nurses has been already mentioned as one of the major obstacles in health system. Regarding nurse population ratio, the reported value varies from less than 0.2 nurses per 1000 population in countries such as Bangladesh, and Liberia to more than 10 nurses per 1000, in countries such as Finland, Norway and Ireland. The direct association was observed between nurse staffing, nurse work environments and nurse education with patient mortality. In Iran, the nurse–bed ratio is estimated to be 0.5:1, while according to the Iranian Ministry of Health and Medical Education the ideal status for this ratio must be 1.8:1 (4).

The above-mentioned work intensifications can be considered as the key factors in nurses’ stress. High level of stress can decrease quality of care and patient safety and is inversely associated with nurses’ work ability (5). Work ability is a complex concept which represents social, mental, and physical capabilities of the employees in the workplace and describes the interactions between mental and physical resources of individuals (6). Work ability is an index of job satisfaction also considered as a key factor related to job quality and security (7). In health-care-related occupations, having high level of work ability is a precondition of performing required tasks under different working conditions (e.g. high number of patients, high work pressure etc.) (8).

In work ability assessments, the ability of workers in completion of the tasks with regard to factors associated with workers mental and physical capabilities are considered (9).

Aging, unhealthy lifestyle, poor physical health, and various diseases have adverse effects on the individuals work ability (10–12). Poor work ability increases job stress and consequently it will diminish the nurses’ quality of work life (5).

The quality of working life represents the employees’ perceptions of their physical and psychological health in relation to their work and the ability of workers to satisfy their important personal needs based on their experiences in the organization (13, 14).

Improved quality of work life and job satisfaction leads to overall satisfaction with the life (14).

QWL of nurses can be affected by social, administrative, managerial, and certain cultural conditions (15). High quality of work life is essential to attract and retain personnel in any organization (16).

The quality of working life represents the person’s perceptions of the organization and can impact the quality of care provided by nurses’ at different level (17). In organizations such as hospital, which nurses constitute the largest proportion of the workforce (18), recruitment and retention of the nursing staff is vital. Therefore, to increase the efficiency of hospital a special attention should be paid to this occupational group (15).

Issues related to employees’ quality of working life influence their job satisfaction and ultimately it affects their intention to leave the job or stay (19). Previous studies have confirmed the effect of employees’ quality of working life on the job satisfaction and ultimately the employees’ intention to leave the job or stay. Any problem and shortcoming in this area can lead to job dissatisfaction and burnout (20, 21). Although nurses represent the largest group of staff working in hospitals and medical care facilities a few studies have focused on the nature of nursing job, regarding quality of working life and their work ability (18). The study reported 74% dissatisfaction with the quality of working life among nurses in Iran (15).

Therefore, given the importance of the abovementioned statements, this study aimed to investigate the relationship between work ability and quality of working life and their associations with background and demographic variables among nurses, working in educational hospitals under the aegis of Tehran University of Medical Sciences, Tehran, Iran.

## Materials and Methods

### Study design

This cross-sectional study was conducted among nurses in the Emergency Unit, Intensive Care Unit (ICU), and Cardiac Care Unit (CCU) of educational hospitals including Shariati, Vali-e-Asr, Farabi, Sina, Rozbeh and Baharlo that are under aegis of Tehran University of Medical Sciences, in 2014. Participation in the study was voluntary.

Data were obtained with the written agreement of the participants. Furthermore, the study was approved by the Ethics Committee of the university.

### Subjects

Due to special importance of ICU, CCU and Emergency Unit, the questionnaires were distributed to all nurses working in these units in hospitals under supervision of Tehran University of Medical Sciences. Volunteers were asked to fill out the questionnaires. Overall, 750 participants returned the completed questionnaires, with a response rate of 93.3%. Of the 750 nurses, 25.71%, 45.71%, and 28.57% were working in emergency unit, ICU, and CCU, respectively.

### Study tools

Data were collected using Persian version of the following instruments: a general questionnaire for collecting data related to demographic and background characteristics, Work Ability Index (WAI) questionnaire for assessing nurses work ability and WRQoL Scale for evaluation of quality of working life.

### WAI questionnaire

Work ability index (WAI) questionnaire has been developed by Finnish Institute of Occupational Health in order to measure individual’s ability to work (22). The questionnaire is consisted of seven items, including current work ability compared with the lifetime best [0–10], work ability in relation to the demands of the job [score range: 2–10], number of current diseases diagnosed by a physician [score range: 1–7], estimated work impairment due to diseases [score range: 1–6], sick leave during the past 12 months [score range: 1–5], personal prognosis of work ability 2 year from now [score range: 1,4, or 7], and mental resources [score range: 1–4]. Each item is evaluated by different number of questions; therefore, the score ranges of items differ with each other. The total score of WAI is calculated by summing up the scores of all items that ranged from 7–49 points. The final WAI score categorized into the following levels: poor [7–27], moderate [28–36], good [37–44], and excellent [45–47]. The Persian version of the questionnaire was provided earlier (22).

### WRQoL Scale

The WRQoL Scale was used in order to collect data related to the quality of working life. This questionnaire is a multidimensional instrument designed in 2007 for assessing the quality of working life in health care system (23). The scale consisted of 24 questions that assess five dimensions of Job and Career Satisfaction (JCS), Working Conditions (WCS), General Well-Being (GWB), Home-Work Interface (HWI), Stress at Work (SAW), and Control at Work (CAW). The participants asked to show their agreement with each question using a five-point likert scale (1=strongly disagree, 5=strongly agree). The final score of WRQoL can be classified into three groups according to the percentile of distribution: 10–30 (low WRQoL), 40–60 (average WRQoL), 70–99 (high WRQoL). The reliability and validity of the questionnaire was examined and confirmed (24).

### Statistical analysis

In the last stage, data were analyzed statistically, using SPSS software version 20 (Chicago, IL, USA). According to the distribution of the variable Pearson and Spearman correlation coefficients, One-way ANOVA, Kruskal-Wallis, T-test and Mann-Whitney were used for the analysis purpose. The *P*-values less than 0.05 were considered as statistically significant.

## Results

Overall, 750 nurses; including 461 female (61.4%) and 289 male (38.6%) participated. The detailed data related to demographic and background characteristics of study population are presented in Table 1. The mean age and work experience of nurses to be 33.1(8.00) and 10.2(7.6) yr, respectively.

**Table 1:** Demographic and background characteristics of study subjects, and mean WRQoL and WAI scores

***Variable***	***Subcategory***	***Frequency (%)***	***Mean WRQoL***	***Mean WAI***
Sex	Male	289(38.6)	78.37(12.36)	36.28(4.49)
	Female	461(61.4)	74.17(14.28)	37.32(4.43)
Age (yr)	22–28	273(36.4)	77.21(13.44)	37.8(4.03)
	29–35	203(27.1)	79.92(12.87)	38.47(4.22)
	36–42	160(21.4)	73.86(14.56)	35.63(4.1)
	43–49	86(11.5)	67.5(9.53)	33.18(4.9)
	50–56	28(3.6)	68(16.46)	35.8(3.11)
Work experience	1–6	284(37.8)	78.11(13.61)	37.98(4.01)
	7–12	187(25)	78.51(12.51)	37.97(4.17)
	13–18	139(18.6)	73.92(14.01)	36.5(4.11)
	19–24	101(13.6)	68.63(14.40)	34.42(4.68)
	25–30	38(5)	71(10.42)	32(4.83)
Unit	Emergency unit	193(25.7)	69.44(13.59)	35.11(5.1)
	ICU	342(45.7)	77.5(13.32)	36.64(4.14)
	CCU	215(28.6)	78.77(12.79)	39(3.52)
Type of employment	Fixed term	252(33.6)	77.12(14.15)	36.48(4.26)
	Contract nurses	498(66.4)	76.25(13.34)	37.52(4.63)
Over-time (hrs/month)	<30	364(48.6)	74.89(14.1)	37.29(4.49)
	30–60	236(31.4)	80.04(12.86)	37.5(4.2)
	60–90	17(2.2)	66.33(0.58)	35.33(3.05)
	>90	133(17.8)	71.88(13.07)	35.08(4.66)
Number of patients under supervision	1–3	300(40)	77.46(13.09)	37.84(4.45)
	4–6	177(23.6)	76.24(14.08)	36.42(3.9)
	7–9	43(5.7)	75.12(15.26)	36.12(6.81)
	10–12	139(18.6)	69.84(12.89)	36.44(4.56)
	13–15	91(12.1)	78.82(14.20)	36.12(4.12)
Number of shifts in a month	10–17	251(33.5)	74.11(14.36)	38.08(4.67)
	18–25	262(35)	77.08(13.13)	36.75(3.51)
	26–33	172(22.9)	76.09(13.54)	36.09(4.69)
	>33	65(8.6)	76.33(14.67)	35.25(5.83)

The mean WAI was obtained 36.9 (range: 7–49) which is in moderate level. Moreover, the mean WRQoL score was estimated 75.7 (range 0–100) which stands in average group (Table 1).

The normality of the study variables were investigated using Kolmogorov-Smirnov test. Of the all study variables, only total WAI, General Well-Being, Job and Career Satisfaction, and total QWL showed normal distribution. Statistical tests revealed significant relationship between total WAI score and total WRQoL (*P*-value=0.001), Stress at Work (*P*-value=0.001), and General Well-Being (*P-*value=0.001). In addition, total WRQoL were positively correlated with mental resources (*P-*value=0.001), number of current diseases diagnosed by a physician (*P*-value=0.02), and work ability in relation to the demands of the job (*P-*value=0.04). A significant positive association was observed between mental resources with Career Satisfaction (*P*-value=0.04), and Working Conditions (*P*-value=0.001), Home-work Interface (*P-*value=0.001), and Well-Being (*P*-value=0.001). Additionally, General Well-Being was associated with work ability in relation to the demands of the job (*P-*value=0.03) and number of current diseases diagnosed by a physician (*P*-value=0.001), (Table 2). Table 3 represents the associations between demographic and background variables and work ability index and its items. The total score of work ability index was conversely associated with nurses’ age and work experience (*P*-value=0.001). However, using One-way ANOVA, a significant difference was observed between WAI score of nurses working in different unit, with the highest score belonging to the CCU (*P*-value=0.001).

**Table 2:** Pearson correlation between WRQoL and WAI

***WAI subitems***	***Current work ability compared with the lifetime best***	***Work abili ty in relation to the demands of the job***	***Number of current diseases diagnosed by a physician***	***Estimated work impairment due to diseases***	***Sick leave during the past 12 months***	***Personal prognosis of work ability 2 yr from now***	***Mental resources***	***Total WAI***
***WRQoL subscales***								
Well-Being	0.97	0.03*	0.001*	0.12	0.09	0.19	0.001*	0.001*
Home-work Interface	0.62	0.56	0.02*	0.55	0.75	0.58	0.001*	0.06
Job Satisfaction	0.41	0.12	0.22	0.76	0.81	0.21	0.001*	0.05
Control at Work	0.35	0.29	0.34	0.52	0.98	0.09	0.06	0.12
Working Conditions	0.77	0.08	0.12	0.65	0.85	0.92	0.001*	0.10
Stress at Work	0.84	0.06	0.26	0.09	0.16	0.15	0.09	0.001*
Quality of Working Life	0.50	0.15	0.07	0.70	0.61	0.20	0.03*	0.001*
WRQoL_total_	0.65	0.04*	0.02*	0.73	0.42	0.16	0.001*	0.001*

**Table 3:** The relation between demographic and background variables with work ability index and its subscales

***Item***		Sex**	***Age***	***Work experience***	Unit**	Employment type**	***overtime***	***Number of shifts***	***Number of patients under supervision***
Current work ability compared with the lifetime best	P	0.28	0.06	0.03*	0.02*	0.91	0.51	0.88	0.24
	r	-	0.15	0.18	-	-	0.05	−0.01	−0.1
Work ability in relation to the demands of the job	P	0.001*	0.57	0.001*	0.42	0.04	0.69	0.05	0.56
	r	-	0.05	0.23	-	-	0.03	−0.16	0.04
Number of current diseases diagnosed by a physician	P	0.13	0.02*	0.001*	0.35	0.12	0.15	0.15	0.17
	r	-	0.19	−0.23	-	-	−0.12	−0.12	−0.11
Estimated work impairment due to diseases	P	0.14	0.07	0.09	0.46	0.25	0.01*	0.000*	0.47
	r	-	−0.15	0.14	-	-	−0.2	−0.31	−0.06
Sick leave during the past 12 months	P	0.01*	0.000*	0.001*	0.001*	0.01*	0.03*	0.01*	0.05
	r	-	−0.36	−0.34	-	-	−0.18	−0.21	−0.23
Personal prognosis of work ability 2 yr from now	P	0.06	0.000*	0.001*	0.001*	0.97	0.21	0.97	0.69
	r	-	−0.29	−0.28	-	-	−0.11	0.002	0.03
Mental resources	P	0.29	0.000*	0.02*	0.07	0.09	0.16	0.36	0.86
	r	-	−0.19	−0.19	-	-	0.12	0.07	−0.01
Total WAI	P	0.07	0.000*	0.001*	0.001*	0.29	0.12	0.05	0.19
	r	-	−0.33	−0.32	-	-	−0.13	−0.16	−0.11

* significant at P-value <0.05

** Using One-way ANOVA, Kruskal-Wallis, T-test, and Mann-Whitney according to the distribution of the variable

The association between demographic and background variables with WRQoL and its dimensions were investigated using different statistical tests. According to presented results in Table 4, total WRQoL score was inversely correlated with age and work experience (*P*-value=0.001) that is in accordance with what was expected. Similar to what was observed for WAI, the total score of QWL was statistically different among nurses in the three study units (*P*-value=0.001).

**Table 4:** The relationships between "demographic and background variables" and "WRQoL and its subscales"

***Demographic and background variables WRQoL subscales***		Sex**	***Age***	***Work experience***	Unit**	Type of employment**	***Overtime***	***Shift numbers***	***Number of patients***
Well-Being	P	0.98	0.01*	0.01*	0.02*	0.13	0.12	0.65	0.07
	R	-	−0.21	−0.22	-	-	−0.13	−0.04	−0.15
Home-work	P	0.28	0.08	0.09	0.15	0.46	0.14	0.62	0.15
Interface	R	-	−0.14	−.014	-	-	0.12	0.04	−0.12
Job Satisfaction	p P	0.37	0.04*	0.03*	0.001*	0.20	0.88	0.33	0.76
	R	-	−.017	−0.18	-	-	0.01	0.08	−0.03
Control at Work	P	0.12	0.27	0.30	0.03*	0.14	0.94	0.33	0.72
	R	-	−0.09	0.08	-	-	0.006	0.08	0.03
Working Conditions	P	0.001	0.02*	0.01*	0.02*	0.60	0.38	0.11	0.47
	R	-	−0.19	−0.21	-	-	−0.07	0.13	0.06
Stress at Work	P	0.80	0.05	0.11	0.08	0.75	0.02*	0.50	0.04*
	R	-	−0.16	−0.13	-	-	−0.18	−0.06	−0.17
Quality of Working Life	P	0.19	0.01*	0.03*	0.001*	0.64	0.19	0.72	0.61
	R	-	−0.19	−0.18	-	-	−0.11	−0.03	−0.04
WRQoL_total_	P	0.18	0.001*	0.001*	0.001*	0.34	0.36	0.58	0.32
	R	-	−0.22	−0.22	-	-	−0.17	0.05	−0.08

* significant at P-value <0.05

** Using One-way ANOVA, Kruskal-Wallis, T-test, and Mann-Whitney according to the distribution of the variable

## Discussion

There is a significant positive association between work ability and QWL in a way that nurses with higher work ability have also higher QWL. The results of this study are consistent with the study that carried out among Canadian nurses and another study (11). By improving components of work ability the employees’ quality of working life and the overall organization productivity would be boosted. Considering that quality of working life is affected by the individuals perceptions of working conditions and capable individuals in the workplace are subjected to low work pressure and occupational stress, enhance the work ability can leads to an increase in quality of working life.

Analysis showed that mean WAI differs in emergency unit, CCU, and ICU. In this regard, the lowest WAI value was belonged to nurses in emergency unit which can be due to the high number of patients, acute status of patients’ health in this unit, high job demand, rapid decision-makings, high job stress and burnout, and the nature of tasks in this unit (25).

In the present research, the mean quality of working life was 75.8 which is close to the value (26) and is not in accordance with the result of another study (27). The differences between result of this study and Almalki et al may be due to differences in the study population, differences in the governing systems in the two communities, and individual differences. Of the reasons for the desirability of quality of working life in this study, we can refer to the open and honest communication, role clarity, participation in decision-making, learning environment and improved clinical competency of nurses. As well as high general feelings of well-being, job satisfaction, control over work, appropriate working conditions, good salary, proper working environment and good organizational policies can lead to high WRQoL (28).

The mean quality of working life was different among nurses of the three understudy working units with the highest WRQoL belonged to nurses in CCU. In CCU, working conditions in a way that nurses is having the opportunity to use their skills and talents. Moreover, in CCU working stressors are lower in comparison to other units. Therefore, nurses have higher WRQoL.

There was a significant and positive association between total WAI score and total quality of working life and General Well-Being. These relationships were reported in some researches (11). In the studies, the work ability was introduced as a critical factor affecting the quality of working life and employees well-being (29). Improved work ability among nurses lead to low work stress and lower job stress leads to higher satisfaction at work and better work-related quality of life (14). positive attitudes of nurses toward their working life and home life, receiving salary and other job benefits regularly, and the nurses’ compatibility with their working conditions can cause higher WRQoL and nurses with higher WRQoL are motivated and therefore have more ability to doing tasks. According to the study results, quality of working life was positively correlated with mental resources and work ability in relation to the demands of the job and it is conversely associated with the current work ability compared with the lifetime best. Mental resources and work ability in relation to the demands of the job are factors that can cause increase in individuals work ability. Quality of working life is affected by the occupational and individual conditions, nurses with high working ability are more compatible with job requirements and have a better view of working conditions; therefore, work ability can enhance the quality of working life.

The inverse relation between the current work ability with the QWL may be because employees may not use their maximum capacities to perform their tasks due to organizational and managerial limitations and lack of participations in decision-makings. This can result in reduced job satisfaction and subsequently decrease of QWL (14). Moreover, lack of opportunity for using personal skills and job promotion can be another reason for the converse relationship between current work ability and WRQoL.

In the emergency unit, there was a significant relationship between General Well-Being, Job and Career Satisfaction, and Home-Work Interface, and total WAI score which is inconsistent with the result of study (30). The Home-Work Interface is pertinent to work-life balance and the employee’s control over their work. Supporting of managers and coworkers increase the home-work interactions and this increase satisfaction with the home life and work life (31).

The work ability index has a significant converse association with age and work experience. In the several study, lower work ability index was reported among older nurses, there was a significant inverse association between WAI, and age and work experience (32–34). In high demanding occupations, individuals’ physical capacity can influence their work ability. Since nursing profession is a highly demanding job, by increase in age their physical capacity decrease. Indeed, by increase in work experience and imposed physical and mental limitations, the work capacity and work ability decreased. Quality of working life was also inversely associated with age and work experience, which is similar to results of the previous researchers (22). This can be due to inappropriate working conditions, high workload, and dissatisfaction with the time that salary paid etc. The results showed a significant association between the working unit and the WAI. Occupational stress as a detrimental factor in the workplace can greatly reduce efficiency and working ability of nurses. Observation of patient death, heavy workload, lack of control over working conditions, working hours and night shifts are among the stressors of nursing that are different in understudy units, therefore the difference in the WAI in different working units could be due to differences in the level of occupational stress.

Increasing WL through financial staff encouragement, retraining and athletic programs to promoting Staff, favorable shift work etc. can improve the QWL that lead to the quality of care improvement.

## Conclusion

There is a two-way interaction between work ability and work-related quality of life. Some individual factors such as age, experience, health status and type of work can affect this interaction by affecting one of these two indices.

## Ethical considerations

Ethical issues (Including plagiarism, informed consent, misconduct, data fabrication and/or falsification, double publication and/or submission, redundancy, etc.) have been completely observed by the authors.
